# Analysis of 14,392 whole genomes reveals 3.5% of Qataris carry medically actionable variants

**DOI:** 10.1038/s41431-024-01656-1

**Published:** 2024-07-17

**Authors:** Amal Elfatih, Chadi Saad, The Qatar Genome Program Research Consortium, Chadi Saad, Said Ismail, Wadha Al-Muftah, Radja Badji, Dima Darwish, Tasnim Fadl, Heba Yasin, Maryem Ennaifar, Rania Abdel-latif, Fatima Alkuwari, Muhammad Alvi, Yasser Al Sarraj, Asmaa Althani, Hamdi Mbarek, Eleni Fthenou, Fatima Qafoud, Eiman Alkhayat, Nahla Afifi, Sara Tomei, Wei Liu, Stephan Lorenz, Najeeb Syed, Hakeem Almabrazi, Fazulur Rehaman Vempalli, Ramzi Temanni, Tariq Abu Saqri, Mohammed husen Khatib, Mehshad Hamza, Tariq Abu Zaid, Ahmed El Khouly, Tushar Pathare, Shafeeq Poolat, Rashid Al-Ali, Omar M. E. Albagha, Souhaila Al-Khodor, Mashael Alshafai, Ramin Badii, Lotfi Chouchane, Xavier Estivill, Khalid Fakhro, Hamdi Mbarek, Younes Mokrab, Jithesh V. Puthen, Karsten Suhre, Zohreh Tatari, Borbala Mifsud, Hamdi Mbarek

**Affiliations:** 1 Hamad Bin Khalifa University, College of Health and Life Science, Genomics and Precision Medicine, Doha, Qatar; 2 Qatar Genome Program, Qatar Foundation, Qatar Science and Technology Park, Innovation Center, Doha, Qatar; 3 William Harvey Research Institute, Queen Mary University London, London, UK; 4 Qatar Biobank for Medical Research, Qatar Foundation, Building 317, Hamad Medical City, Doha, Qatar; 5 Sidra Medicine, Integrated Genomics Services, Out-Patient Clinic, Doha, Qatar; 6 Sidra Medicine, Applied Bioinformatics Core—Integrated Genomics Services - Research Branch, Doha, Qatar; 7 Sidra Medicine, Biomedical Informatics—Research Branch, Doha, Qatar; 8 College of Health and Life Sciences, Hamad Bin Khalifa University, Education City, Doha, Qatar; 9 Sidra Medicine, Maternal and Child Health Program, Doha, Qatar; 10 College of Health Sciences, Qatar University, Doha, Qatar; 11 Molecular Genetics Laboratory, Hamad Medical Corporation, Doha, Qatar; 12 Departments of Genetic Medicine, Microbiology and Immunology, Weill Cornell Medicine-Qatar, Doha, Qatar; 13 Quantitative Genomics Laboratories (qGenomics), Barcelona, Spain; 14 Sidra Medicine, Human Genetics Department, Doha, Qatar; 15 Sidra Medicine, Computational Genomics and Data Science Laboratory, Doha, Qatar; 16 Bioinformatics Core, Weill Cornell Medicine-Qatar, Education City, Doha, Qatar; 17 Sidra Medicine, Clinical Research Center, Doha, Qatar

**Keywords:** Consanguinity, Genetic counselling

## Abstract

Arabic populations are underrepresented in large genome projects; therefore, the frequency of clinically actionable variants among Arabs is largely unknown. Here, we investigated genetic variation in 14,392 whole genomes from the Qatar Genome Program (QGP) across the list of 78 actionable genes (v3.1) determined by the American College of Medical Genetics and Genomics (ACMG). Variants were categorized into one of the following groups: (1) Pathogenic (P), (2) Likely pathogenic (LP), and (3) Rare variants of uncertain significance with evidence of pathogenicity. For the classification, we used variant databases, effect predictors, and the disease-relevant phenotypes available for the cohort. Data on cardiovascular disease, cancer, and hypercholesterolemia allowed us to assess the disease-relevant phenotype association of rare missense variants. We identified 248 distinct variants in 50 ACMG genes that fulfilled our criteria to be included in one of the three groups affecting 1036 genotype-positive participants of the QGP cohort. The most frequent variants were in TTN, followed by RYR1 and ATP7B. The prevalence of reportable secondary findings was 3.5%. A further 46 heterozygous variants in six genes with an autosomal recessive mode of inheritance were detected in 200 individuals, accounting for an additional 1.4%. Altogether, they affect 5% of the population. Due to the high consanguinity rate in the QGP cohort (28% in spouses and 60% in parents), P and LP variants both in genes with dominant and recessive inheritance are important for developing better treatment options and preventive strategies in Qatar and the Arabic population of the Middle East.

## Introduction

The advance in next-generation sequencing technologies over the last two decades has promoted the use of genome/exome sequencing in research and clinical testing and made patients’ genomic data more accessible. Investigating pathogenic variants in human genomes assists in diagnosing genetic diseases and in improving the guidelines and prevention policies, leading to precise management [1]. Pathogenic variants in disease-causing genes that are not directly related to the patient’s primary concern but may be medically relevant can also be identified by genome /exome sequencing [2, 3].

The American College of Medical Genetics and Genomics (ACMG) published a policy in 2017 that emphasized the value of notifying patients before genome/exome sequencing about the possibility of secondary findings. Laboratories are recommended to report specific types of variants in 59 highly penetrant genes with significant clinical impact [4]. In 2021, the ACMG released an updated policy statement for reporting secondary findings (SF) [5]. Seventy-eight genes have been included in the SF v3.1 list for reporting secondary results, intending to update the gene list annually [6]. The last update (SF v 3.2) was published in June 2023, including 81 genes [7].

The reported frequency of secondary findings in populations with mostly European ancestries ranged between 0.3 and 2.34% using the 59 ACMG gene list [8–12]. However, the frequency of secondary findings in Arab populations is not as extensively studied, even though their population structure, due to the high consanguinity, makes these populations vulnerable to genetic diseases. Such high rate of consanguinity means that genetic diseases are among the leading causes of pediatric mortality in Arab populations [13], and it is expected to increase the prevalence of diseases associated with the 78 ACMG genes. We previously reported the frequency of the medically actionable variants using the ACMG reporting guidelines in Qatar using 6045 whole genome sequencing (WGS) data from the Qatar Genome Program (QGP). We found that 2.3% of Qatari participants carry a pathogenic or likely pathogenic variant in one of the 59 ACMG genes [14].

Due to the bias in variant submission and clinical annotation towards variants present in European-descent populations, using the ACMG reporting guidelines can miss population-specific pathogenic variants in underrepresented populations. In this study, we used a pipeline that is appropriate for underrepresented populations[15] to estimate the frequency of the pathogenic and likely pathogenic medically actionable variants in the SF v3.1 list of 78 ACMG genes using WGS data from the population-based cohort of the QGP (*n* = 14,392). Moreover, we aimed to assess a set of variants with predicted pathogenic effects in genes associated with cancer and cardiovascular disease (CVD) and examine their disease evidence in the QGP cohort.

## Materials and methods

### Cohort description and bioinformatics analysis

This study was based on a cohort of 14,392 Qatari participants from the population based QGP [16]. Ethical approval was provided by the institutional review board of the Qatar Biobank (QBB) (Protocol no. QF-QGP-RES-PUB-002). A detailed description of the recruitment process and collection of phenotypic data by the QGP and the QBB was previously reported [16, 17]. A signed consent was obtained from all included participants by the QBB, and WGS data used in the current study was obtained from the QGP as previously described [17].

All samples were sequenced on Illumina HiSeq X instruments (Illumina, San Diego, CA, USA) to a target average depth of coverage of 30x. Quality control of the produced FastQ files was performed using FastQC (v0.11.2). The read mapping, variant calling and joint variant calling were performed using Sentieon’s DNASeq pipeline [18] v201808.03 (Sentieon, San Jose, CA, USA), following the BWA-GATK Best Practice Workflow and using the GRCh38/hg38 reference genome. The produced gVCF were jointly called to produce one multisample VCF file (msVCF) for the whole cohort. We then trimmed the msVCF to retain only the 78 medically actionable genes as recommended by the ACMG SF v3.1 [19]. Multi-allelic variants were then decomposed and normalized, and only passing-filter variants with minor allele frequency (MAF) <2% were kept. Remaining variants were annotated with Ensembl Variant Effect Predictor (VEP, release 106.1) [20], using ClinVar (version 20221105) [21] and HGMD (version HGMD_Pro_2022.3) [22] databases. We also used the AutoPVS1 (v2.0) [23] and REVEL (v1.3) [24] tools for in silico pathogenicity prediction.

Since VEP provides prediction for each transcript of the gene, we selected the MANE transcript. If no MANE transcript was available for a gene, we selected the transcript with the most severe prediction according to VEP consequence rank. Finally, if multiple transcripts had the same rank, we selected the longest one.

### Classification and Interpretation of ACMG Variants

We retained variants that overlapped with the 78 ACMG genes reported in SF v3.1[19] and had a MAF <2% in the QGP data. We classified the variants based on ClinVar review and clinical significance criteria [25]. Variants that were annotated as Benign (B) or Likely Benign (LB) in ClinVar were discarded from the rest of the analysis. The rest of the variants were assessed to see if they fulfill the criteria for one of the following groups: (1) Validated Pathogenic (P), (2) Likely Pathogenic (LP), and (3) rare Variants of Uncertain Significance with evidence of pathogenicity (pVUS).

### Pathogenic (P) list

To generate a list of high-confidence pathogenic variants, we consolidated a list of previously reported and validated P variants based on the variant classification in ClinVar only or both in ClinVar and HGMD. We divided the P list into two sub-classes, P1 and P2. All variants annotated as Pathogenic/Likely Pathogenic (P/LP) in ClinVar and as a disease-causing mutation (DM) in HGMD were included in the P1 list. Variants with conflicting annotation of pathogenicity between ClinVar and HGMD were excluded from this sub-category.

Variants in the P2 list were defined as variants annotated as P/LP and having two or more gold stars in the ClinVar database clinical significance status. The two or more gold stars criterion means that expert panels reviewed variants, multiple submitters reported the variant’s pathogenicity without conflict in their interpretation, or variant classification was obtained from a practice guideline [25].

### Likely pathogenic (LP) list

Variants with potentially deleterious effects were classified into the LP list. We divided variants in the LP list into two sub-classes: LP1 and LP2. In LP1, we described loss of function (LOF) variants, which are either insertions/deletions, nonsense, frameshift, or splice site variants, and were annotated as PVS1 (very strong) using the AutoPVS1 automated classification tool. We further filtered these LOF variants to keep only rare (MAF < 0.1% in QGP and gnomAD) and high impact (VEP impact = “HIGH”) variants.

Missense variants in ClinVar (annotated with conflicting interpretation of pathogenicity or as variants with uncertain significance (VUS)) and novel variants (defined as not reported in dbSNP, gnomAD, ClinVar and HGMD) were investigated to be included in the LP2 list. We filtered for rare variants (MAF < 0.1% in QGP and gnomAD) that were carried by more than one genotype‐positive participants (GPP), were predicted to be pathogenic using the REVEL *in-silico* prediction tool (score > 0.7) and had a CVD or cancer disease evidence. These variants were annotated using the VarSome API [26] and we used the final verdict interpretation according to the VarSome API combinatory rules. Only variants annotated as P or LP using VarSome API were included in the LP2 list.

### Rare variant of uncertain significance with evidence of pathogenicity (pVUS) list

Rare reported and novel missense variants with the same characteristics as LP2 variants but lacking the P or LP classification entry by the VarSome API were classified as pVUS.

### Frequency of genotype positive participants (GPPs)

We determined the number of GPPs, individuals who carried at least one allele of the variants in the three categories (P, LP, pVUS). We calculated the frequency of GPPs and the 95% CI per disease category using a one proportion Z-test.

### Phenotypic assessment for disease evidence

All QGP participants underwent clinical and physical examinations, and their medical history was taken using an approved and standardized questionnaire conducted by trained nurses. The questionnaires captured information about the participants’ and their families’ medical records.

The study cohort was deeply phenotyped, featuring cardiovascular, cancer, and hypercholesterolemia disease evidence. CVD evidence was defined as having abnormalities in the participants’ ECG records, self-reported information about heart diseases, heart revascularization (bypass, angioplasty, or coronary atherectomy), and parents’ or siblings’ history of heart diseases.

Similarly, evidence of cancer was obtained, including self-reported data about participants’ or parents’ history of cancer and record of tumor-related surgery. In addition, we used breast and colon cancer screening as disease evidence when conducted on participants younger than the recommended gender-specific age for screening by the Primary Health Care Corporation (PHCC) in Qatar [27, 28]. Finally, lipid-related measurements, including total cholesterol, high-density lipoprotein (HDL), and low-density lipoprotein (LDL) levels, and detailed information on hypercholesterolemia diagnosis and treatment options were also obtained.

We extracted the above-mentioned phenotypic data related to CVD and cancer for all participants in the QBB. We then identified unique variants in CVD- or cancer-associated ACMG genes that were carried by at least two GPPs with supporting phenotypic information related to CVD or hypercholesterolemia or by at least one GPP with personal or family cancer evidence. We assessed the disease phenotype-association of each such variant.

### Statistical analysis

We used R version 4.2.2 (2022-10-31) for all statistical analyses. The disease phenotype association of LP2 and pVUS variants was tested using Fisher’s exact test. We described the association by the OR and 95% CI, and we used the *p* values: *p < 0.05, **p < 0.01, ***p < 0.001, ****p < 0.0001, to express the level of statistical significance for CVD or cancer disease evidence in the QGP cohort.

## Results

### Variants in the 78 ACMG genes in the Qatari Population

**Fig. 1:**
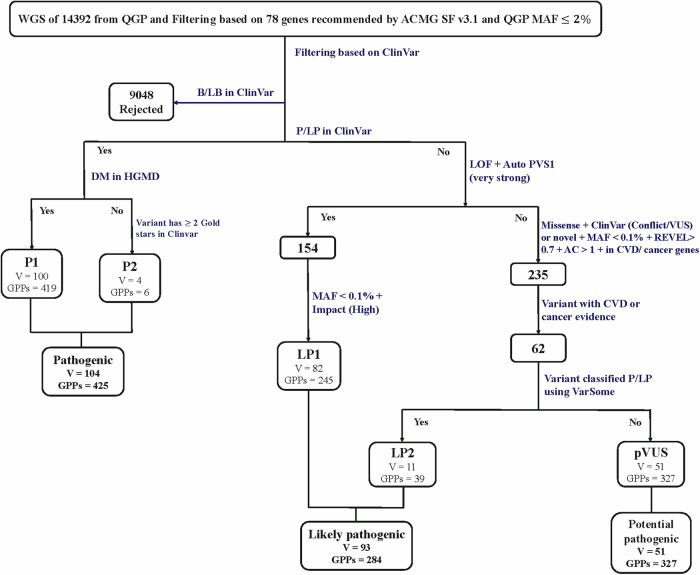
Flow chart of the pipeline for actionable variant identification in 78 ACMG genes. WGS whole genome sequencing, QGP Qatar Genome Program, B benign, LB likely benign, P pathogenic, LP likely pathogenic, DM disease-causing, HGMD Human Gene Mutation Database, LOF loss of function, VUS rare variants of uncertain significance with evidence of pathogenicity, AC allele count, MAF minor allele frequency, CVD cardiovascular diseases, V variants, GPPs genotype-positive participants.

**Fig. 2:**
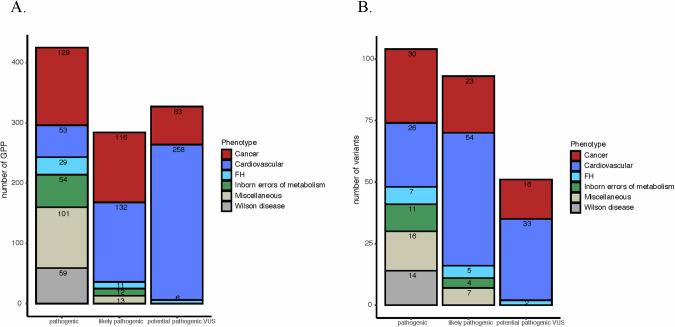
Variants and participants in the Qatar genome program reported lists. **A** Total number of genotype positive participants carrying variants in the QGP across the three lists. **B** Total number of variants in the QGP across the three lists based on 78 ACMG genes. GPP genotype positive participants, FH familial hypercholesterolemia.

The QGP data set analyzed in this study comprised 14,392 whole genomes of the Qatari population. There were 9048 distinct variants of the 78 ACMG genes that were annotated as B/LB in the ClinVar database and were rejected from subsequent analyses (Fig. 1). The remaining variants were assessed to see if they can be classified into one of three lists P, LP, and pVUS. We identified 248 distinct variants in 50 ACMG genes that fulfilled our criteria to be included in one of our lists (Supplementary Table 1, Additional File 1). In total, 1,036 GPPs (7.2% of QGP cohort) (Fig. 2A) carried at least one of the P, LP, or pVUS variants (Fig. 2B). All variants were identified in dominant genes of the ACMG secondary findings gene list, except for 46 variants (14 in *ATP7B*, 9 in *MUTYH*, 8 in *GAA*, 7 in *BTD*, 5 in *TRDN*, and 3 in *RPE65*), which were carried by 200 heterozygous GPPs responsible for recessive conditions. Among our three categories, *TTN* variants were the most frequent (41 variants, 166 GPPs), followed by variants in *RYR1* (16 variants, 62 GPPs) and variants in *ATP7B* (14 variants, 59 GPPs).

The analysis revealed 104 pathogenic variants identified based on ClinVar and HGMD disease entries fulfilling the P list criteria. Most P list variants (100 variants) were classified in the P1 list (419 GPPs). Only four are in P2 (6 GPPs), which had two gold stars or more in ClinVar but were not annotated as DM in HGMD.

The LP1 list described 82 variants carried by 245 GPPs (1.7% of the QGP cohort). These 82 were LOF variants with PVS1 strength evidence of pathogenicity in 24 genes, where LOF is a known disease-causing mechanism; therefore, according to ACMG/AMP standards and guidelines [29, 30], they are likely to be pathogenic. Furthermore, in the LP2 list, we described 11 variants. These were filtered from 235 novel variants (not present in dbSNP, gnomAD, ClinVar and HGMD), or reported variants (VUS/ conflicting interpretation of pathogenicity in ClinVar) that had a REVEL score >0.7, were rare (QGP MAF < 0.1%), were found in more than one QGP participants (AC > 1), and in CVD- and cancer-related genes. We identified 11 variants carried by 39 GPPs (HET:39 and HOMO:0) that were annotated as P or LP using the VarSome API and showed cardiovascular or cancer disease evidence in the QGP cohort. These 11 variants were found in 10 genes responsible for dominant conditions.

Lastly, in the pVUS list, 51 variants had either a conflicting interpretation of pathogenicity in ClinVar, were annotated as VUS or were novel, had MAF < 0.1% and REVEL score >0.7, and had cardiovascular or cancer disease evidence in the QGP cohort but were not annotated as P or LP using the VarSome API tool. These 51 variants were found in 21 genes responsible for dominant conditions and carried by 327 GPPs (HET: 325 and HOMO: 2).

### Phenotypes of LP2 and pVUS genotype-positive participants

After applying the filters on missense variants that were novel or had ClinVar entry of conflicting interpretation of pathogenicity/VUS, we identified 235 variants, carried by 1155 GPPs, which were found in genes known to be associated with cardiovascular or cancer phenotypes. We reviewed the phenotypic records of 1155 GPPs to identify cardiovascular or cancer disease evidence in the QGP cohort. We identified 366 GPPs, carrying 62 variants, with cardiovascular or cancer disease evidence (Additional File 2 and 3).

11 of these variants (in 39 GPPs) were annotated as P or LP by the VarSome API and classified in the LP2 list. Only Two variants c.454 G > A (p.Ala152Thr) in *SDHD* and c.185 C > G (p.Pro62Arg) in *BRCA1* showed significant disease association (*P* = 0.02) (Supplementary Table 1, Additional File 2).

An additional 327 GPPs carrying 51 CVD- or cancer-related variants were found to have disease evidence; however, were not annotated as P or LP using the VarSome API. We found significant association with the relevant disease phenotypes for 11 variants carried by 72 GPPs in the QGP cohort.

Four tumor-associated variants in 21 GPPs were reported in the pVUS list. These had significant association with personal or family history of cancer-related phenotypes in the QGP cohort. Two GPPs carrying *MSH2* c.2684 C > T (p.Pro895Leu) had personal breast cancer history (*p = 0.017, OR = 117.8, 95% CI = 1.5), 13 GPPs carrying *MSH6* c.3100 C > T (p.Arg1034Trp) (*p = 0.013, OR = 17.2, 95% CI = 1.4) or *TSC2* c.2158 A > G (p.Lys720Glu) (*p = 0.019, OR = 12.9, 95% CI = 1.16) had personal history of tumor-related surgeries, and 6 GPPs carrying *MSH2* c.641 G > T (p.Arg214Ile) (**p = 0.006, OR = 24.1, 95% CI = 2.2) reported parent’s history of cancer, or parent’s cause of death was cancer (Supplementary Table 1, Additional File 3).

48 individuals carried cardiovascular disease-related pVUS variants (c.2477 T > C (p.Leu826Ser) in *DSG2*, c.223 C > A (p.Gln75Lys) in *MYL3*, c.5065 C > A (p.Arg1689Ser) in *MYH7*, c.326 T > G (p.Val109Gly) in *LMNA*, c.77035 A > C (p.Asn25679His) in *TTN*, and c.10255 T > C (p.Phe3419Leu) in *RYR2*) and reported cardiovascular phenotypes (self-reported history of personal and parents heart disease, and parents’ cause of death was related to heart disease) (Supplementary Table 2, Additional File 2). Additionally, we also identified 3 GPPs carrying the c.1145 G > T (p.Gly382Val) variant in the *LDLR* gene with significant association with lab records indicating high LDL levels (*p = 0.02, OR = 21.4, 95% CI = 1.1) (Supplementary Table 3, Additional File 2). A detailed description of the QGP participants with cardiovascular or cancer phenotypes in the pVUS category, along with the VarSome API annotation of these variants, were described in Supplementary Tables 1, 2, 3, and Additional File 3.

### Distribution of actionable variants among ACMG disease categories

**Fig. 3:**
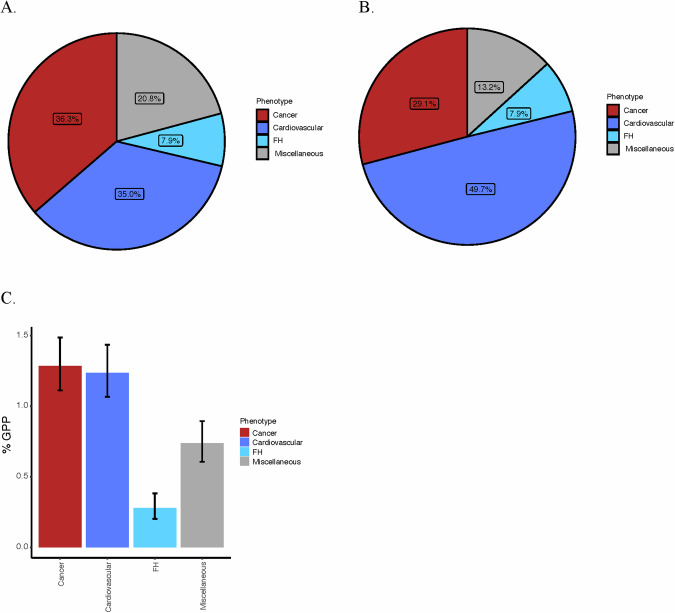
The distribution of actionable variants based on ACMG disease categories. **A** Distribution of genotype positive participants carrying actionable variants based on 78 ACMG gene-associated phenotypes. **B** Distribution of actionable variants among 78 ACMG gene- associated phenotypes. **C** Frequency of genotype positive participants carrying actionable variants based on 78 ACMG gene-associated phenotypes. GPP genotype positive participants, FH familial hypercholesterolemia.

Following the ACMG guidelines for reporting secondary findings, only P, or LP variants for dominant conditions or biallelic P/LP variants for recessive diseases should be reported. 509 GPPs (HET: 508 and HOMO: 1) carrying 151 variants, including 67 P and 84 LP variants, met the ACMG threshold for reporting (Fig. 3A, B). All these variants are associated with phenotypes inherited dominantly.

Across actionable variants in P and LP lists, the highest number of variants (57%; 87/151) and with the highest frequency of GPPs (43%; 218/508) was identified in genes related to cardiovascular predisposition (including familial hypercholesterolemia) (Table 1). Many of these were LOF variants in the newly added cardiomyopathy causing *TTN* gene (31 variants in 96 GPPs). 36% of the GPPs harbor variants of cancer-associated genes mostly related to Hereditary breast/ovarian cancer (42%; 78/185). Actionable variants in both cardiovascular disease-related and cancer-related genes affect more than 1% of the population and we also observed a high frequency of GPPs with variants in *RYR1* (12%; 62/508) associated with Malignant hyperthermia (Fig. 3C).

**Table 1:** Number of actionable variants identified in 78 ACMG genes from the Qatar genome program.

Gene	Inheritance	Variants	GPP	HET	HOMO	Phenotype
**Genes related to cancer phenotypes**						
APC	AD	1	3	3	0	Familial adenomatous polyposis
RET	AD	3	7	7	0	Familial medullary thyroid cancer
BRCA1	AD	9	27	27	0	Hereditary breast and/or ovarian cancer
BRCA2	AD	8	36	36	0	
PALB2	AD	1	15	15	0	
SDHD	AD	1	2	2	0	Hereditary paraganglioma-pheochromocytoma 1.0 syndrome
SDHB	AD	2	2	2	0	
TMEM127	AD	1	1	1	0	
BMPR1A	AD	2	5	5	0	Juvenile Polyposis Syndrome
TP53	AD	1	3	3	0	Li–Fraumeni syndrome
MSH2	AD	1	4	4	0	Lynch syndrome
MSH6	AD	4	16	16	0	
PMS2	AD	3	20	20	0	
PTEN	AD	3	4	4	0	PTEN hamartoma tumor syndrome
TSC2	AD	2	24	23	1	Tuberous sclerosis complex
VHL	AD	2	16	16	0	von Hippel-Lindau syndrome
**Genes related to cardiovascular phenotypes**						
PKP2	AD	3	3	3	0	Arrhythmogenic right ventricular cardiomyopathy
DSP	AD	1	1	1	0	
DSC2	AD	2	2	2	0	
DSG2	AD	3	3	3	0	
RYR2	AD	1	1	1	0	Catecholaminergic polymorphic ventricular tachycardia
TNNT2	AD	3	3	3	0	Dilated cardiomyopathy
LMNA	AD	2	3	3	0	
FLNC	AD	2	5	5	0	
TTN	AD	31	96	96	0	
DES	AD	2	3	3	0	
COL3A1	AD	4	4	4	0	Ehlers-Danlos syndrome, vascular type
LDLR	SD	7	30	30	0	Familial hypercholesterolemia
APOB	AD	5	10	10	0	
MYH7	AD	5	12	12	0	Hypertrophic cardiomyopathy
MYBPC3	AD	2	3	3	0	
TNNI3	AD	3	10	10	0	
MYL3	AD	1	1	1	0	
KCNQ1	AD	6	20	20	0	Long QT syndrome types 1 and 2
KCNH2	AD	1	1	1	0	
SCN5A	AD	3	7	7	0	Long QT syndrome 3, Brugada syndrome
**Genes related to miscellaneous phenotypes**						
HNF1A	AD	2	4	4	0	Maturity-onset of diabetes of the young
RYR1	AD	16	62	62	0	Malignant hyperthermia
TTR	AD	2	40	40	0	Hereditary TTR amyloidosis

*GPP* genotype positive participants, *HET* heterozygous, *HOMO* homozygous, *AD* autosomal dominant, *SD* semidominant.

Only five individuals had more than one P or LP variant in two genes: two GPPs harboring predisposition to two cancer phenotypes, one GPP had reported variants in two CVD-related genes, one GPP was predisposed to cancer and Hereditary TTR amyloidosis, and lastly one GPP was found to have cancer and Malignant hyperthermia related variants (Table 2).

**Table 2:** Genotype positive participants with reported variants in multiple ACMG genes.

Gender	Age	Variant 1	Phenotype 1	Variant 2	Phenotype 2
F	39	*BRCA2* c.4211_4215del (p.Ser1404Ter)	Hereditary breast and/or ovarian cancer	*PTEN* c.379 G > A (:p.Gly127Arg)	PTEN hamartoma tumor syndrome
F	35	*PALB2* c.2479del (p.Thr827HisfsTer24)	Hereditary breast and/or ovarian cancer	*RET* c.2410 G > A (p.Val804Met)	Familial medullary thyroid cancer
F	35	*TNNI3* c.434 G > A (p.Arg145Gln)	Hypertrophic cardiomyopathy	*TTN* c.67495 C > T (p.Arg22499Ter)	Dilated cardiomyopathy
F	25	*BRCA1* c.4787 C > A (p.Ser1596Ter)	Hereditary breast and/or ovarian cancer	*TTR* c.424 G > A (p.Val142Ile)	Hereditary TTR amyloidosis
M	28	*VHL* c.154 G > T (p.Glu52Ter)	von Hippel-Lindau syndrome	*RYR1* c.8655del (p.Trp2886GlyfsTer42)	Malignant hyperthermia

*F* female, *M* male, *HET* heterozygous.

## Discussion

In this study, we investigated the prevalence of secondary findings variants in the QGP cohort according to the ACMG SF v3.1 list. We first analyzed the genomic data of 14,392 QGP participants and interpreted the variants using a newly developed pipeline that is more adequate for underrepresented populations. As a result, we found that 7.2% of the QGP participants carry at least one pathogenic, likely pathogenic, or rare variant of uncertain significance with evidence of pathogenicity. We identified 104 P variants reported either in ClinVar only or in the ClinVar and HGMD databases and 82 LOF LP1 variants that led to truncated proteins. Review of the available phenotypic records related to CVD and cancer and using the VarSome API revealed 11 P or LP variants that showed positive association with the personal or family history of 39 GPPs, therefore these variants were found likely to be pathogenic (LP2). Our study detected P or LP variants in 5% (709/14392) of the QGP individuals. 3.5% of the QGP cohort carried reportable actionable variants according to the ACMG guidelines, and an additional 1.5% carried one of 46 heterozygous variants in six genes (*MUTYH*, *ATP7B*, *BTD*, *GAA*, *RPE65*, and *TRDN*) associated with diseases, which are inherited in an autosomal recessive fashion. The GPP rate was especially high for the *MUTYH* and *ATP7B* genes, which indicate a high carrier rate of MUTYH-Associated Polyposis and Wilson disease in the Qatari population. Similarly to other countries in the region, Qatar is known for its unique population structure characterized by a high consanguinity rate and increased prevalence of rare genetic diseases. A study that was published recently by Abdu et al. showed that the prevalence of consanguineous marriage among married couples was 62.6% [30]. In the current study, 28% of participants had consanguineous spouses, and 60% had consanguineous parents. Although those heterozygous variants did not meet the reporting criteria of ACMG secondary findings [29], informing patients of the disease risks in their offspring or relatives is still necessary due to the high frequency of consanguinity in the QGP cohort.

The 3.5% frequency of secondary findings was consistent with a recently published report from an Asian population (3.4%) [15] and higher than other reports (1.8% in the Taiwanese population [31], 2.1% in the Lebanese population [32], and 2.8% in the European population [33]). This difference could be partially attributed to the expanded ACMG gene list we used for screening for secondary findings, as the medically actionable variants of SF v.3.1 genes accounted for 32% (164/508) of GPPs carrying actionable P or LP variants in our cohort. Most existing reports were based on the previous gene lists of 59/73 genes. Based on 59 genes, we previously reported that the frequency of actionable variants in Qatar using 6045 WGS from the QGP was 2.3% [14].

The highest frequency of GPPs with P or LP variants was found in genes associated with cardiovascular diseases, especially dilated cardiomyopathy; this result is predominantly due to the high number of LOF variants in the *TTN* gene and it is consistent with the high prevalence of this disease in Qatar, and in the Arab region [34, 35]. Of note, we recently examined the pathogenicity of the *LMNA* c.326 T > G (p.Val109Gly) variant (*p = 0.015, OR = 4.16, 95% CI = 1.25) described in the pVUS list. We found that it was significantly associated with cardiovascular disease in the previously assessed 6045 WGS data from the QGP, and functional validation of the variant using zebrafish revealed abnormalities in heart rate, structure, and function [36]. Additionally, we observed a high frequency of GPPs with P and LP variants associated with Malignant hyperthermia (8.7%), MUTYH-associated polyposis (8.5%), and Wilson’s disease (8.3%). This finding is consistent with previous observations that the Arabic population has a high prevalence of Wilson’s disease [37, 38], which is mostly caused by pathogenic *ATP7B* variants. In a recent study by Al Jumah et al., 56 Saudi patients with Wilson disease were investigated, and 50% of the cohort were found to have a pathogenic variant in the *ATP7B* gene [39]. Genetic screening for pathogenic *ATP7B* variants could help with the diagnosis of Wilson’s disease. Its diagnosis currently relies on sensible clinical suspicion and a study conducted by Prashanth et al., which evaluated 307 patients diagnosed with Wilson’s disease, found that there was a two-year mean delay in diagnosing the condition due to poor clinical suspicion attributed to atypical presentation and the involvement of neurological, hepatic and eye symptoms [40]. Thus, further studies on rare variants in the *ATP7B* gene will be needed to guide laboratories whether those variants need to be reported and to improve the diagnostic yield of Wilson’s disease.

A recent study published by Aarabi et al. [41] investigated the frequency of reportable copy number variations (CNVs) in the ACMG SFv3.2 genes among 10,959 patients who were tested by chromosomal microarray analysis. The study identified 57 ACMG SFv3.2 genes for which sufficient evidence supports reporting of partial or complete copy number losses as secondary findings. Overall, CNVs in SFv3.2 genes were detected in 32/10,959 (0.29% or 1 in 343) patients from the cohort. Given that CNVs were not considered in this study, and based on the above mentioned study, the frequency of actionable variants could be underestimated in the Qatari population.

Our study’s main limitation is that we could not evaluate disease evidence across all tested genes due to the lack of phenotypic information. The available disease-related phenotypes only allowed us to investigate variants in CVD-related genes, including hypercholesterolemia, and cancer. Investigating the genetic basis of such severe conditions in this understudied population offers a unique opportunity to identify novel disease-causing variants, and subjects and family members at risk. Therefore, further genetic counseling and early intervention could help to improve the life quality of QGP participants. This study has an exciting potential for developing better treatment options and preventive strategies in Qatar and the Arabic population of the Middle East.

## Supplementary information

Additional file 1

Additional file 2

Additional file 3

## Data Availability

The data of this study is available from the QGP and QBB. Still, restrictions apply to the availability of these data, which were used under license QF-QGP-RES-PUB-002 for the current research and are not publicly available. Data are, however, available from the authors upon request and with permission of the QBB institutional review board.
